# A stochastic simulation model to determine the sample size of repeated national surveys to document freedom from bovine herpesvirus 1 (BoHV-1) infection

**DOI:** 10.1186/1746-6148-3-10

**Published:** 2007-05-18

**Authors:** Lea Knopf, Heinzpeter Schwermer, Katharina DC Stärk

**Affiliations:** 1 Swiss Federal Veterinary Office, Schwarzenburgstrasse 155, CH-3003 Bern, Switzerland

## Abstract

**Background:**

International trade regulations require that countries document their livestock's sanitary status in general and freedom from specific infective agents in detail provided that import restrictions should be applied. The latter is generally achieved by large national serological surveys and risk assessments. The paper describes the basic structure and application of a generic stochastic model for risk-based sample size calculation of consecutive national surveys to document freedom from contagious disease agents in livestock.

**Methods:**

In the model, disease spread during the time period between two consecutive surveys was considered, either from undetected infections within the domestic population or from imported infected animals. The @Risk model consists of the domestic spread in-between two national surveys; the infection of domestic herds from animals imported from countries with a sanitary status comparable to Switzerland or lower sanitary status and the summary sheet which summed up the numbers of resulting infected herds of all infection pathways to derive the pre-survey prevalence in the domestic population. Thereof the pre-survey probability of freedom from infection and required survey sample sizes were calculated. A scenario for detection of infected herds by general surveillance was included optionally.

**Results:**

The model highlights the importance of residual domestic infection spread and characteristics of different import pathways. The sensitivity analysis revealed that number of infected, but undetected domestic herds and the multiplicative between-survey-spread factor were most correlated with the pre-survey probability of freedom from infection and the resulting sample size, respectively. Compared to the deterministic pre-cursor model, the stochastic model was therefore more sensitive to the previous survey's results. Undetected spread of infection in the domestic population between two surveys gained more importance than infection through animals of either import pathway.

**Conclusion:**

The model estimated the pre-survey probability of freedom from infection accurately as was shown in the case of infectious bovine rhinotracheitis (IBR). With this model, a generic tool becomes available which can be adapted to changing conditions related to either importing or exporting countries.

## Background

In 2002, Hadorn and co-workers showed how to combine confidence obtained in a survey conducted previously and the risk of disease introduction thereafter [1]. To quantify the residual confidence at the beginning of the following survey, quantitative and qualitative risk assessment methods were used. In this deterministic model the potential risk of undetected infected herds spreading the infectious agent in-between two surveys in the population under study was not considered. This limited the approach to non-highly contagious diseases, because the number of infected herds is nearly constant in-between two surveys.

**Figure 1:**
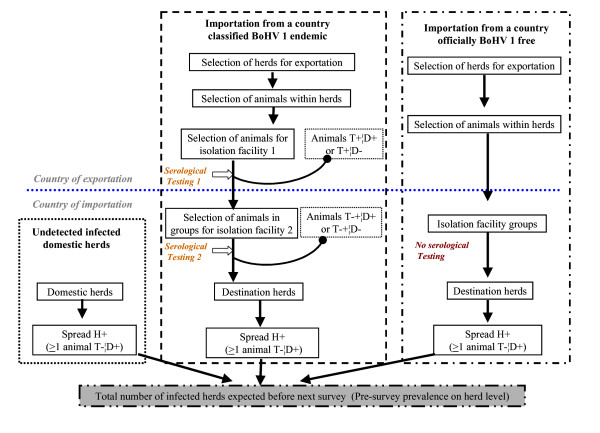
**Basic model structure and cattle import pathways**. Basic structure of the stochastic simulation model to estimate the pre-survey probability of freedom from infection as applied to BoHV-1. Three main pathways of infectious agent introduction into the susceptible domestic population were considered. + = infected, - = uninfected, D = infection status of animal, T = diagnostic test status of animal, H = herd status

Based on the agricultural agreement from 1999, Annex 11, and the bilateral agreements with the EU new import regulations for cattle have been implemented in Switzerland on 1st July 2004 [2]. These regulations lead to a distinction of sanitary categories of breeding cattle import to Switzerland and hence different import pathways depicted in Figure 1.

The main objective of this study was to incorporate the spread of infection in the model to obtain an improved estimate of the pre-survey probability of freedom (pre-SPF), and, in consequence, the minimal required sample size for national surveys to demonstrate freedom from more rapidly spreading disease agents. The second objective was to consider changes in the EU-import regulations and third, to investigate implications of disease awareness and general surveillance in Switzerland. This stochastic model should be of a generic type and adaptable to a wide range of infectious agents. The use and parameterization of the model were illustrated on the example of infectious bovine rhinotracheitis (IBR).

## Methods

The model structure was based on the current import regulations and possible infection links to the domestic population (Figure 1). International guidelines of import risk analysis [6] were followed throughout the model construction. The model was realised in three sub-models and a summarizing part: The first sub-model was dedicated to the spread of undetected infection within the domestic population; two sub-models accounted for infection via imported animals, either originating from countries that are officially free from the infective agent or from countries where the infective agent and the disease is present. The outputs of the three sub-models were cumulative probability distributions of numbers of infected herds. In the summarizing part of the model, the resulting numbers of infected farms of each sub-model were summed up per iteration to the final simulation output. From 1'000 iterations, a probability distribution of the updated herd prevalence prior to the survey was derived. This prevalence distribution was used to derive the point estimate pre-SPF from infection in the national cattle population and, subsequently, to calculate the sample size for the planned survey. For simulation and distribution details we refer to Table 1.

Uncertainty and variability were incorporated using probability distributions of parameters and Monte Carlo simulation (@Risk, Palisade Tools, Version 4.5.2.). The correlation of input parameters with output parameters was based on Spearman rank correlation coefficient calculations (significance level of p ≤ 0.05). The following paragraphs elucidate the detailed structure and calculations steps of each sub-model, on the example of BoHV-1.

### Undetected domestic infection and domestic spread of the infectious agent

Undetected residual infection and potential spread between herds in the domestic population was included in the model. The range for the residual herd prevalence (*HP*) was sampled from a beta distribution. The distribution was defined by the previous survey size (*N*) and detected positive herds in the previous survey (*s*), beta (*s*+1;*N-s*+1).

The potential between-survey spread was interpreted as a multiplicative transmission factor which would approximately predict annual changes in *HP *due to disease spread between herds. This between-survey spread factor (BSSF), was empirically derived for annual periods, between two consecutive surveys in years (*k *and *k+1*) from the means (*μ*) of *HP *beta distributions:

BSSFk+1=μbeta(HPk+1)μbeta(HPk)
 MathType@MTEF@5@5@+=feaafiart1ev1aaatCvAUfKttLearuWrP9MDH5MBPbIqV92AaeXatLxBI9gBaebbnrfifHhDYfgasaacH8akY=wiFfYdH8Gipec8Eeeu0xXdbba9frFj0=OqFfea0dXdd9vqai=hGuQ8kuc9pgc9s8qqaq=dirpe0xb9q8qiLsFr0=vr0=vr0dc8meaabaqaciaacaGaaeqabaqabeGadaaakeaacqWGcbGqcqWGtbWucqWGtbWucqWGgbGrdaWgaaWcbaGaem4AaSMaey4kaSIaeGymaedabeaakiabg2da9maalaaabaacciGae8hVd0MaemOyaiMaemyzauMaemiDaqNaemyyaeMaeiikaGIaemisaGKaemiuaa1aaSbaaSqaaiabdUgaRjabgUcaRiabigdaXaqabaGccqGGPaqkaeaacqWF8oqBcqWGIbGycqWGLbqzcqWG0baDcqWGHbqycqGGOaakcqWGibascqWGqbaudaWgaaWcbaGaem4AaSgabeaakiabcMcaPaaaaaa@50B3@

All annual BSSF values were derived from data of the disease-specific national surveys from 1994–2005 and descriptive statistics provided minimum, maximum and most likely values (median) for a beta pert distribution. A worst case scenario was assumed, which did not allow for a decrease in *HP *(BSSF ≥1 in all runs).

To account for disease awareness and general surveillance, the option for a binomial detection probability was included for newly infected herds. Newly infected herds were defined as the number of herds that were infected by domestic spread during the time between two surveys. In the case of BoHV-1, studies from the Netherlands [7] on unvaccinated, certified BoHV-1 free herds indicated that only about 80% of new infections on herd level result in perceived "major" outbreaks with potential of further spread to additional herds. In the other 20% of infected herds (*M*) the within herd prevalence (*whp*) of infection would be very low and the likelihood of immediate spread to other cows and herds is less likely. The infection theoretically might even die out due to random culling. It was assumed that farmers and veterinary practitioners would notice or ignore new outbreaks on herd level, depending on the patterns of within herd spread and prevalence of clinical signs of the disease, which is also related to virulence of virus strain. In the case of a noticed outbreak, control measures would be implemented and further spread from herd to herd halted. The probability of undetected infected herds (*Q*) was defined as follows:

p(Q=x)=(MQ)p(nond)Q*(1−p(nond))M−Q
 MathType@MTEF@5@5@+=feaafiart1ev1aaatCvAUfKttLearuWrP9MDH5MBPbIqV92AaeXatLxBI9gBaebbnrfifHhDYfgasaacH8akY=wiFfYdH8Gipec8Eeeu0xXdbba9frFj0=OqFfea0dXdd9vqai=hGuQ8kuc9pgc9s8qqaq=dirpe0xb9q8qiLsFr0=vr0=vr0dc8meaabaqaciaacaGaaeqabaqabeGadaaakeaacqWGWbaCcqGGOaakcqWGrbqucqGH9aqpcqWG4baEcqGGPaqkcqGH9aqpdaqadaqaauaabaqaceaaaeaacqWGnbqtaeaacqWGrbquaaaacaGLOaGaayzkaaGaemiCaa3aaeWaaeaacqWGUbGBcqWGVbWBcqWGUbGBcqWGKbazaiaawIcacaGLPaaadaahaaWcbeqaaiabdgfarbaakiabcQcaQiabcIcaOiabigdaXiabgkHiTiabdchaWjabcIcaOiabd6gaUjabd+gaVjabd6gaUjabdsgaKjabcMcaPiabcMcaPmaaCaaaleqabaGaemyta0KaeyOeI0Iaemyuaefaaaaa@52A0@

Where *Q *is the number of undetected, infected herds, *M *is the number of newly infected herds and *p(nond) *is 1-probability of detection. The probability of *Q *was then multiplied with *M *to obtain the number of domestic undetected infected herds within the country before the next survey.

### Infection via direct import

Only animals from countries with sanitary conditions comparable to Switzerland were admitted for direct import (Figure 1). This import pathway included no serological testing, therefore the probability of importing infected animals was derived from the animal level prevalence and a binomial selection process for export in the country of origin. Data from previous surveys in the country of origin were used to estimate the expected residual *HP *as described above. Otherwise standard requirements of EU regulations (64/432/EEC) were used as a basis to define the *HP *beta distribution (*N *> 2300, *s *= 0).

Based on the prevalence of infection in imported animals (*prev*) and the number of animals imported (*n*), the probabilities of selecting 1 or 2 or 3...to x infected animals (*m*) for import were calculated. The total number of imported infected animals (*m*_*total*_) from a specific country and for the whole import pathway was obtained by Eq. 3:

mtotal=∑m≥0n(nm)prevm*(1−prev)n−m*m
 MathType@MTEF@5@5@+=feaafiart1ev1aaatCvAUfKttLearuWrP9MDH5MBPbIqV92AaeXatLxBI9gBaebbnrfifHhDYfgasaacH8akY=wiFfYdH8Gipec8Eeeu0xXdbba9frFj0=OqFfea0dXdd9vqai=hGuQ8kuc9pgc9s8qqaq=dirpe0xb9q8qiLsFr0=vr0=vr0dc8meaabaqaciaacaGaaeqabaqabeGadaaakeaacqqGTbqBdaWgaaWcbaGaemiDaqNaem4Ba8MaemiDaqNaemyyaeMaemiBaWgabeaakiabg2da9maaqahabaWaaeWaaeaafaqaaeGabaaabaGaemOBa4gabaGaemyBa0gaaaGaayjkaiaawMcaaiabdchaWjabdkhaYjabdwgaLjabdAha2naaCaaaleqabaGaemyBa0gaaOGaeiOkaOYaaeWaaeaacqaIXaqmcqGHsislcqWGWbaCcqWGYbGCcqWGLbqzcqWG2bGDaiaawIcacaGLPaaadaahaaWcbeqaaiabd6gaUjabgkHiTiabd2gaTbaakiabcQcaQiabd2gaTbWcbaGaemyBa0MaeyyzImRaeGimaadabaGaemOBa4ganiabggHiLdaaaa@599C@

where *prev *= HP * *whp*

Cattle are imported throughout the whole year. The disease transmission potential of infected import animals needed therefore to be adjusted (BSSF_adj_). The remaining time within the domestic population until the next survey was considered by a time weighting factor (*tw*_*x*_) on a monthly basis. The starting point in the year was set just after the Swiss national surveys that are usually carried out in March. Origin and average numbers and of monthly imported animals from the last three years were used to determine the monthly import probabilities *p(imp)*:

BSSF_adj _= BSSF * *tw*_x_* *p(imp)*_*t *= *x*_

The expected total number of infected animals of Eq. 3 was then multiplied with each monthly BSSF_adj _value to consider the spread until the next survey accurately. The sum of all monthly estimates provided the total number of infected animals through direct import. It was assumed that each infected animal would result in one infected domestic herd (worst case scenario).

### Infection via indirect import

This import pathway was applied for cattle originating from countries or regions having a lower sanitary status, thus considered not officially free from the infectious disease agent or in our case BoHV-1 (Figure 1). Animals of the indirect pathway were supposed to be kept in quarantine or isolation facilities in groups and to undergo two serological testing rounds (Figure 1). If an animal tested positive in the quarantine of the export country it was excluded. If at least one animal tested positive in the second testing round in the isolation facility in Switzerland, the whole group was rejected. In consequence, the conditional probability that an infected group was released to the destination farm was the probability of the group to have at least one infected animal given that all animals were negative reactors, also in the second serological testing round.

Only animals that tested negative in the first testing round were admissible for importation. The probability to introduce a certain number *x *of infected animals *(m*_*start*_*) *into each isolation facility of group size *(g) *in Switzerland was given by the probability of selecting animals having a false negative test result in the exporting country. Therefore the test sensitivity of the first serological test in the export country (*Se1*) was included:

p(mstart=x)=(gmstart){(prev*(1−Se1))mstart*(1−(prev*(1−Se1)))}g−mstart
 MathType@MTEF@5@5@+=feaafiart1ev1aaatCvAUfKttLearuWrP9MDH5MBPbIqV92AaeXatLxBI9gBaebbnrfifHhDYfgasaacH8akY=wiFfYdH8Gipec8Eeeu0xXdbba9frFj0=OqFfea0dXdd9vqai=hGuQ8kuc9pgc9s8qqaq=dirpe0xb9q8qiLsFr0=vr0=vr0dc8meaabaqaciaacaGaaeqabaqabeGadaaakeaacqWGWbaCdaqadaqaaiabd2gaTnaaBaaaleaacqWGZbWCcqWG0baDcqWGHbqycqWGYbGCcqWG0baDaeqaaOGaeyypa0JaemiEaGhacaGLOaGaayzkaaGaeyypa0ZaaeWaaeaafaqaaeGabaaabaGaem4zaCgabaGaemyBa02aaSbaaSqaaiabdohaZjabdsha0jabdggaHjabdkhaYjabdsha0bqabaaaaaGccaGLOaGaayzkaaWaaiWaaeaadaqadaqaaiabdchaWjabdkhaYjabdwgaLjabdAha2jabcQcaQmaabmaabaGaeGymaeJaeyOeI0Iaem4uamLaemyzau2aaSbaaSqaaiabigdaXaqabaaakiaawIcacaGLPaaaaiaawIcacaGLPaaadaahaaWcbeqaaiabd2gaTnaaBaaameaacqWGZbWCcqWG0baDcqWGHbqycqWGYbGCcqWG0baDaeqaaaaakiabcQcaQmaabmaabaGaeGymaeJaeyOeI0YaaeWaaeaacqWGWbaCcqWGYbGCcqWGLbqzcqWG2bGDcqGGQaGkdaqadaqaaiabigdaXiabgkHiTiabdofatjabdwgaLnaaBaaaleaacqaIXaqmaeqaaaGccaGLOaGaayzkaaaacaGLOaGaayzkaaaacaGLOaGaayzkaaaacaGL7bGaayzFaaWaaWbaaSqabeaacqWGNbWzcqGHsislcqWGTbqBdaWgaaadbaGaem4CamNaemiDaqNaemyyaeMaemOCaiNaemiDaqhabeaaaaaaaa@7FC2@

In view of the required minimum isolation facility period in Switzerland, the model accounted for the probability of transmission of an undetected infection within the isolation group and detection by the second serological test before the group was admitted to the destination farm. The number of potentially infected animals within the isolation facility group at the end (*m*_*end*_) of the isolation period were obtained by inclusion of the transmission probability *p(trans) *and the number of infected animals within the group at start of the isolation (*m*_*start*_):

*m*_*end *_= (*g *- *m*_*start*_) * (*p*(*trans*) * (*g - m*_*start*_)/g) + *m*_*start*_

The probability that all animals of the isolation facility group were not infected and still test negative *(allD*-|*T*--*) *at the end of the period was further dependant on the test sensitivity (*Se2*) and specificity (*Sp2*) of the second testing round:

p(allD−|T−−)=Sp2g−mend*(mendg*(1−Se2))mend
 MathType@MTEF@5@5@+=feaafiart1ev1aaatCvAUfKttLearuWrP9MDH5MBPbIqV92AaeXatLxBI9gBaebbnrfifHhDYfgasaacH8akY=wiFfYdH8Gipec8Eeeu0xXdbba9frFj0=OqFfea0dXdd9vqai=hGuQ8kuc9pgc9s8qqaq=dirpe0xb9q8qiLsFr0=vr0=vr0dc8meaabaqaciaacaGaaeqabaqabeGadaaakeaacqWGWbaCdaqadaqaaiabdggaHjabdYgaSjabdYgaSjabdseaejabgkHiTiabcYha8jabdsfaujabgkHiTiabgkHiTaGaayjkaiaawMcaaiabg2da9iabdofatjabdchaWnaaDaaaleaacqaIYaGmaeaacqWGNbWzcqGHsislcqWGTbqBdaWgaaadbaGaemyzauMaemOBa4MaemizaqgabeaaaaGccqGGQaGkdaqadaqaamaalaaabaGaemyBa02aaSbaaSqaaiabdwgaLjabd6gaUjabdsgaKbqabaaakeaacqWGNbWzaaGaeiOkaOYaaeWaaeaacqaIXaqmcqGHsislcqWGtbWucqWGLbqzdaWgaaWcbaGaeGOmaidabeaaaOGaayjkaiaawMcaaaGaayjkaiaawMcaamaaCaaaleqabaGaemyBa02aaSbaaWqaaiabdwgaLjabd6gaUjabdsgaKbqabaaaaaaa@5DF8@

Therefore the probability that the group contained at least one infected, not detected animal at the end of the isolation facility period was 1- *p(all D*-|*T*--*)*. In a final step, the resulting number of infected groups was summed up and then multiplied by BSSF_adj_, as described in the direct import pathway. In the indirect import pathway, all animals from one infected group were assumed to be delivered to the same farm and to infect it.

### Pre-survey prevalence and pre-survey probability of freedom

In each of the 5,000 iteration runs of the model, the expected number of infected herds of each pathway was summed up to a final number of infected herds before the next survey. The resulting pre-survey prevalence in the domestic population (N = 50'000 herds) was expressed as a probability distribution. With regard to the internationally required *HP*-threshold level and the defined confidence (Table 1), the pre-SPF from infection was derived directly from the *HP*'s cumulative probability distribution as the proportion of the sampled values that lay beyond the threshold *HP*. Starting from the overall level of confidence required (γ) and pre-SPF estimate, the required confidence level *z *(1 - type-I error) for the consecutive survey can be calculated by Eq.8 of Hadorn et al. [1]:

z=(γ−preSPF)(1−preSPF)
 MathType@MTEF@5@5@+=feaafiart1ev1aaatCvAUfKttLearuWrP9MDH5MBPbIqV92AaeXatLxBI9gBaebbnrfifHhDYfgasaacH8akY=wiFfYdH8Gipec8Eeeu0xXdbba9frFj0=OqFfea0dXdd9vqai=hGuQ8kuc9pgc9s8qqaq=dirpe0xb9q8qiLsFr0=vr0=vr0dc8meaabaqaciaacaGaaeqabaqabeGadaaakeaacqWG6bGEcqGH9aqpdaWcaaqaamaabmaabaacciGae83SdCMaeyOeI0IaemiCaaNaemOCaiNaemyzauMaem4uamLaemiuaaLaemOrayeacaGLOaGaayzkaaaabaWaaeWaaeaacqaIXaqmcqGHsislcqWGWbaCcqWGYbGCcqWGLbqzcqWGtbWucqWGqbaucqWGgbGraiaawIcacaGLPaaaaaaaaa@45F5@

### Data on bovine herpes virus 1

BoHV-1 data used as input for the parameterization of the stochastic model originated from the surveillance programme, import and trading statistics, literature as well as expert opinion on infection dynamics (Table 2). The eradication of clinical cases related to BoHV-1 was accomplished in 1992. Currently, Switzerland is officially free from BoHV-1. Since 1994, Switzerland has conducted annual serological surveys to substantiate claims of freedom from BoHV-1. From 2002 onwards, quantitative and qualitative RA, together with the information from the last year's survey, provided the basis for risk-based sample size calculations for consecutive surveys as described by Hadorn et al. [1]. Detailed data on conducted or planned Swiss BoHV-1 surveys from 2002–2007 are shown in Table 2. The model did not account for cattle that were imported for slaughter, because spread of infectious agents from them to the domestic population was assumed to be unlikely.

## Results

The model consisted of three separate sub-models and a summarising part: Consideration of 1) disease spread from undetected infected domestic herds; 2) introduction of infection due to import from a disease free country with an equivalent livestock health status; 3) introduction of infection due to import from a non disease free country with a lower livestock health status, and 4) a summarizing sub-model calculating the pre-SPF and the required sample size for the next survey.

### Sensitivity analysis

The rank correlation of input parameters showed that the pre-survey prevalence and hence the pre-SPF was most correlated with the remaining baseline *HP *within Switzerland (r = 0.97) and modestly with values of the between survey spread factor (BSSF) (r = 0.20). Infection through direct import from countries officially free from BoHV-1 was most correlated with the *HP *in the country of origin (r = 0.76) and to lesser extent with the number of imported animals and the assumed *whp *for infected herds in the country of origin (r = 0.46 and 0.32, respectively). Estimates of infection caused by indirect import from countries not officially free from BoHV-1 was also dependant on the number of imported animals (r = 0.36). Increasing isolation facility group size in the indirect import pathway was negatively correlated with the model outcome pre-SPF (r = -0.14). The impact of the value of the between survey spread factor, which was adjusted for import seasonality (BSSF_adj_), on both, direct and indirect import pathway outcomes, was negligible (r<0.1).

The residual domestic *HP *prior to the next survey accounted for 96.97% (95% CI 96.63–97.31%) of the pre-survey prevalence estimate on average, whereas the mean contribution of the direct import pathway was 1.55% (1.40–1.70%) and 0.29% (0.24–0.33%) of the indirect import pathway, respectively. The inclusion of a binomial detection probability increased the correlation of pre-survey prevalence with the inland prevalence to r = 0.99. Second high-ranking contribution to the pre-survey prevalence was the number of undetected infected herds defined by the binomial detection probability (r = 0.73). All other model parameters ranked thereafter showed negligible correlations of r<0.1. For the outcome of the individual direct and indirect import pathways, no relevant change in parameter ranking or correlation coefficients was observed.

### Exploration of model structure and behaviour

#### Herd prevalence

**Figure 2:**
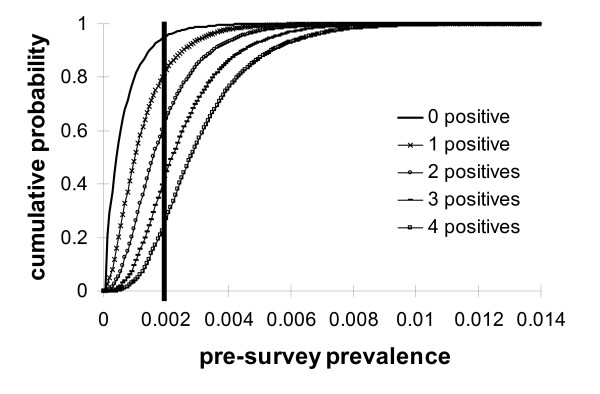
**Cumulative probability distribution of pre-survey prevalence with increasing numbers of positive samples detected**. Effect of increasing residual herd prevalence (*HP*) on the pre-survey prevalence estimates prior to the consecutive survey at a threshold HP of 0.2% (vertical black bar). Pre-survey herd prevalence was estimated using cumulative herd prevalence beta-distributions defined by a fixed sample size (N = 2400) and increasing numbers of positive samples

Figure 2 presents the increase of the predicted model pre-survey prevalence distribution in relation to an increasing residual *HP *within the importing country. Step-wise increments of the residual *HP *were simulated by increasing discrete numbers of positive samples (s = 0 to 5) from a previous survey of a fixed sample size (N = 2400). This sample size accounted for possible ranges of imperfect test characteristics and was sufficient to prove a *HP *< 0.2% at a 99% confidence level. Starting without infected herds, one more infected herd detected in the previous survey implicated a mean of 29.5 more infected herds before the next survey and a loss in pre-SPF of 18.2% per additional positive survey finding. Each increase in *HP *by 0.1% shortly after the last survey lead to 75.6 more infected herds.

#### BSSF

BSSF values ranged from 1.00 to 3.96, (median 1.03). The mean pre-survey prevalence and the pre-SPF displayed a linear relationship with BSSF. Each one-unit-increase in BSSF reduced the baseline pre-SPF by 11.22%. Where BSSF = 1 meant no spread, BSSF = 2, infected herds doubled through spread in-between two surveys. If a decline in the number of infected herds between two surveys was accepted (BSSF and BSSF_adj _values <1 to ≥1), lower pre-survey *HP *in the sub-model outputs were observed. The difference in *HP *between the two scenarios, namely BSSF values ≥1 and BSSF values <1 to ≥1, had different significance levels depending on the infection pathway: Resulting *HP *of undetected infected domestic herds p = 0.011; infected herds from import from a country with an equivalent livestock health status p = 0.015; import from a country with a lower livestock health status p = 0.14 (no significant difference).

#### Numbers of imported animals

The model predicted one more newly infected domestic herd per 4,765 directly imported animals. In the case of indirect import, the restrictive import requirements limited the introduction of infected groups and animals such that a mean of 9207 indirectly imported animals or 1315 groups of 7 animals respectively, resulted in one newly infected herd only. If we applied the same scenarios for indirect and direct import pathways, namely that each infected animal (instead of an infected group) lead to one additional infected domestic herd, the outcome of the indirect import pathway increased by a factor of 2.85.

#### Within herd prevalence

The sub-model "direct import" was run with an increasing *whp *using fixed values from 0.1 to 0.9 in steps of 0.2. A *whp *of 0.9 implicated at most 1.84 additional infected herds (mean = 0.19) assuming n = 500 directly imported animals in this pathway. The application of sampled *whp *values from a uniform distribution, ranging from 0.1 to 0.9 returned a maximum of 1.67 (mean = 0.11) additional infected herds.

### Application using Swiss BoHV-1 survey data

**Figure 3:**
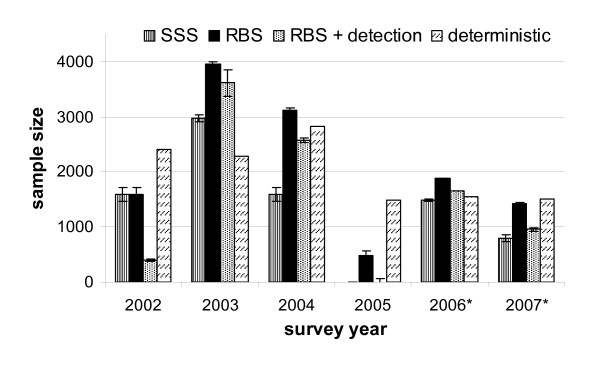
**Comparative sample size of serological surveys derived from the pre-SPF**. Comparative sample sizes for the follow-up survey derived from the model output pre-survey probability of infection freedom (pre-SPF). Standard sample sizes (SSS), risk-based sample sizes (RBS), with or without detection of newly infected herds, were used to define the herd prevalence (*HP*) of the previous survey. Ninety-five percent confidence intervals of pre-survey probability of infection freedom and hence resulting sample sizes, were obtained by exact binomial confidence intervals based on the proportion of iterations with values < 0.2 and < 0.1%, respectively, to the total number of iterations performed. Sample sizes were calculated with Survey Toolbox^® ^assuming a population of 50,000 herds, 99% herd sensitivity for diagnostic testing and a threshold *HP *of 0.2% and 0.1%, respectively.

The input values derived from the Swiss BoHV-1 surveillance programme and comparative results of the deterministic precursor model [1] and of the developed stochastic model outputs are listed in Table 2. About half of the decrease in the pre-SPF of the deterministic model was due to import of animals, mainly direct import (data not shown). The other half of the decrease in pre-SPF of the deterministic model accounted for a fixed margin for potential spread and qualitative RA aspects. In the stochastic model, the estimation of the residual *HP *considered infection from both the domestic and the foreign cattle population. Under these conditions, the residual domestic *HP *and the outcome of the previous survey became more influential in determining the pre-SPF. In consequence, important pre-SPF differences between the model results for the years 2003 and 2006 were observed (Table 2). These years were preceded by surveys that detected an infected domestic herd. If a scenario with a detection probability for newly infected herds was applied, the decline in pre-SPF was less distinct (Table 2). The difference in pre-SPF with a detection probability was highly significant (p < 0.001) if compared to the values of the deterministic model or to the stochastic model (using either risk-based or standard sample sizes for calculation). The consequences for the minimal required sample sizes and their 95% confidence intervals of the different scenarios using the stochastic model are depicted in Figure 3. In the situation where the previous survey outcome was negative, the stochastic model predicted significantly higher pre-SPF compared to the estimate from the deterministic model (Table 2, years 2002, 2004–2005 and 2007).

## Discussion

The stochastic model presented here is a comprehensive combination of quantitative RA components and disease transmission components to simulate both, the spread of disease within a country and the probability of introduction of the infectious agent. The flexible structure of the stochastic model reflecting the current import regulations allows including various additional domestic or import-related risk factors, such as short term animal movements or infection via livestock commodities. Such factors can be incorporated directly as additional numbers of infected herds to the summarizing sub-model or – if necessary – can be incorporated as separate spreadsheet models. Information on numbers of infected herds or animals and the cause of infection are normally available from statutory case reporting or diagnostic laboratory records.

The first objective of the study, to regard the consideration of infection spread between two consecutive surveys was achieved by the introduction of BSSF and BSSF_adj _for spread from domestic herds and imported animals, respectively. Compared to the deterministic precursor model, where a preliminary fixed value for the potential spread between surveys was included, the pre-SPF of the stochastic model reflected disease dynamics more realistically. In the context of repeated surveys, the model highlighted the potential importance of residual infections within the domestic population of a country or region officially free from the disease and the infectious agent, respectively. Especially when a country has to deal with rapidly spreading infections that are characterized by latent phases or that might show only mild to in-apparent clinical signs.

With the introduction of a novel spread factor, explicit transmission modelling in terms of a SIR model was avoided. Depending on the infectious agent's epidemiology, the model allowed in the worst case scenario only for an increase of *HP *(BSSF ≥1) between two consecutive surveys, whereas the alternative scenario allowed also for a decrease of *HP *between two surveys (BSSF values < 1 to ≥1). The alternative scenario modelled a natural extinction course of infection in the case of chronic or slowly spreading infectious agents (e.g. bovine leucosis virus), whereas the worst case scenario was more adapted to rapidly spreading infections with latent disease phases followed by reactivation (e.g. BoHV-1). The different model outputs of the two scenarios above were related to the influence of BSSF on the outcome of the individual infection pathways, as highlighted in the sensitivity analysis of model input parameters.

To include the changes in the EU-import regulations, the BSSF was adjusted for seasonality of import and categories of export countries, this realistically reflected the complex dynamics of cattle populations and the trading patterns between two surveys. However, sound data are lacking to consider spread from clustered outbreaks or clustered sources of infection. As an approximation to clustered spread from imported animals to domestic herds, the release of animals from the two import pathways to their destination farms were treated differently: Delivery of single animals versus groups of animals in the direct import pathway and the indirect import pathway, respectively. Because of the moderate contribution of the indirect pathway to the final model output, a scenario where individual animals were released each to different destination herds did not significantly affect the model's final outcome. The distinct separation of release patterns per import pathway category was therefore a pragmatic approach to address the second objective of the study, but release of groups or single animals in reality may also occur in the other pathway. Consistent data on grouping and delivery patterns to destination farms are expected in future, as the new import regulations have been applied only since July 2004. In the future the model could be adapted to account for heterogeneous spread (clustering of outbreaks), or different susceptibilities of herds for infection (e.g. due to potential risk factors such as larger herd size or intense animal movements, known from other countries).

The high influence of residual domestic *HP *on the pre-SPF in the model was not surprising (Figure 2): The adoption of risk-based national surveys presumably shifted the mean of the assumed residual *HP *beta-distribution to higher values due to a smaller sample size. Model outputs pointed out a significant increase in pre-SPF exceeding the estimates of the deterministic model (Table 2), when using hypothetical standard sample sizes to define input *HP*.

With regard to the third objective of the study, the option of including a detection probability of newly infected herds in terms of general surveillance showed significantly higher pre-SPF estimates, too. In the case of rapidly spreading infections disease notification and interventions to halt the course of an epidemic are to be expected.

In the case of parameterization of the model with BoHV-1 we tested and excluded the scenario that included aspects of general surveillance and detection of outbreaks, although the outputs suggested lower *HP *and higher Pre-SPF estimates. The only estimate for detection probability for BoHV-1 available was derived from a study on Dutch dairy herds that might have different herd sizes and management strategies [4,5]. However, more investigations on detection probabilities of BoHV-1 outbreaks of countries officially free from the infectious agent would lead to reconsideration of this scenario.

For BoHV-1 data and surveys, it was demonstrated, how to account for various factors affecting the health status and disease dynamics of a national cattle population between two surveys to substantiate freedom from infection. The present model was also successfully applied to national risk-based surveillance for freedom from bovine leucosis virus, the causative infectious agent of enzootic bovine leucosis, a more chronic disease and less contagious infection (data not shown).

The number of resulting infected domestic herds through imported BoHV-1 positive animals per pathway was in line with findings of European risk assessments [3]. E.g. the contribution of infection through animals originating from countries not BoHV-1 free was considered very small, because of the high likelihood of detection of positive animals during pre-export quarantine. Whereas introduction of positive animals from countries BoHV-1 free was mainly dependant on the export country's survey results or the assumed *HP*. Experts also mentioned sanitary conditions during up-loading and transport to be an additional source of infection, but in the present study detailed data from export countries on transport conditions were unavailable.

Our BSSF validation suggested realistic values if compared to reports from other BoHV-1-free countries or regions that conduct full surveys. Reporting from Denmark, Bolzano and Austria indicated that spread of infection between herds was very limited, usually 0–1 secondary cases [3].

## Conclusion

The stochastic model is an improved approach to determine risk-based sample sizes for repeated surveys to substantiate claims of freedom on contagious diseases. With this model, a generic tool becomes available which can be adapted to the changing conditions either in the importing and exporting countries or to the disease dynamics. This tool is applicable to the risk-based survey design of a wide range of infectious diseases.

## Authors' contributions

LK drafted the manuscript. LK, HS and KDCS conceived the study. LK and HS developed the basic structure of the simulation model together and LK elaborated the individual modules. KDCS advised on the simulation study and aspects of surveillance. LK generated and analysed the data. All authors critically assessed the study and read and approved the final manuscript.

## Figures and Tables

**Table 1:** Description of baseline model input parameter values and sources. Input values or distributions of parameters used to model the import probability and domestic spread of BoHV-1 in Switzerland between annual serological surveys.

**Parameter**	**Values or distribution as used in @RISK simulation**	**Reference**
Population size	See Table 2	Swiss animal movement database
Sample size last survey	See Table 2	[8]
Number of herds tested positive	See Table 2	[8]
Threshold herd prevalence	0.1–0.2% a)	64/432/ECC 2004/78/EC
Between survey spread factor (BSSF)	= RiskPert(1;1.03;3.96)	derived as explained in the text
Probability of detection	= RiskBinomial(number of newly infected herds; 0.2)	[7]
Number and seasonality of animals imports	records per month and country	Swiss Fed. Custom Statistics
Herd prevalence countries not BoHV-1 free	= RiskUniform(0.1;0.3)	[9, 10]
Herd prevalence countries BoHV-1 free	= RiskBeta(1;2326)	64/432/ECC
Within herd prevalence countries not free	= RiskUniform(0.1;0.5) b)	[11, 12]
Within herd prevalence countries free	= RiskUniform(0.1;0.9) b)	[13–15]
Test sensitivity 1	= RiskUniform(0.87;0.99)	[10, 16, 17]
Test specificity 1	= RiskUniform(0.95;1)	
Isolation facility group size	= 7	[18]
Transmission probability	= RiskUniform(0.25;0.35)	expert opinion
Test sensitivity 2	= RiskUniform(0.2;0.9175)	[3], expert opinion
Test specificity 2	= 1 c)	[3], expert opinion

a) changes in regulation see Table 2

b) dependant on vaccination policy and coverage or eradication programme strategies

c) any positive screening test result would be subject to confirmatory testing

**Table 2:** Results and parameterization of the stochastic model with Swiss national survey data on BoHV-1. Detailed information on BoHV-1 surveys conducted or planned from 2002–2007 in Swiss cattle and pre-survey probability of freedom estimates using deterministic and stochastic model approaches. Sample sizes of previous surveys in the stochastic model: (SSS) standard sample size calculation or (RBS) sample size calculation as described by Eq. 8.

Input values	**2002**	**2003**	**2004**	**2005**	**2006 **^a)^	**2007 **^a)^
Population size (number of cattle herds)	50,591	48,231	48,790	46,574	46,574	46,300
Allowed threshold herd level prevalence	0.001	0.001	0.001	0.002^b)^	0.002	0.002
Number of positive herds detected in previous survey	0	1	0	0	1	0
Sample size of previous survey RBS	632	2400	2274	2828	1430	1550
Sample size of previous survey SSS	4650	4650	4650	2325	2325	2325
*n *direct imports from BoHV-1-free countries	^c)^	^c)^	^*c*)^	155	976	552
*n *indirect imports from BoHV-1-infected countries	500^d)^	8 ^*e*)^	496	1145	388	658
*n *indirect imports, national eradication of BoHV-1 accomplished but not officially free					344	680
Remaining pre-SPF from the last survey, deterministic model [1]	0.8910	0.9094	0.8371	0.8947	0.8201	0.8618
Remaining pre-SPF from the last survey, stochastic model ^f) ^(RBS)	0.9516	0.4958	0.7826	0.9740	0.5866	0.8320
Remaining pre-SPF from the last survey, (RBS) and bin. detection probability of 0.8	0.9856	0.6406	0.872	0.9936	0.7312	0.9336
Remaining pre-SPF from the last survey, stochastic model (SSS)	0.9516	0.8106	0.9516	0.9958	0.8104	0.9514

a) planned surveys that are not carried out yet or analysis not completed

b) adaptation of Swiss regulation to European animal disease surveillance regulation

c) Swiss regulation on cattle import changed in July 2004, specific distinction between animal imports originating from EU countries officially free or not free from certain diseases

d) due e) qualitative risk-assessment only. The import number was adjusted to *n *= 500 for the model input to obtain comparable results with the qualitative estimate

e) import stop due to foot and mouth disease outbreaks in Europe 2001

f) the stochastic model developed and investigated in this publication
